# The impact of a pharmacist intervention on post-discharge hypnotic drug discontinuation in geriatric inpatients: a before-after study

**DOI:** 10.1186/s12877-023-04139-y

**Published:** 2023-07-04

**Authors:** Lorenz Van der Linden, Julie Hias, Astrid Liesenborghs, Karolien Walgraeve, Pieter Van Brantegem, Laura Hellemans, Koen Milisen, Jos Tournoy

**Affiliations:** 1 Hospital Pharmacy Department, University Hospitals Leuven, Herestraat 49, 3000 Leuven, Belgium; 2 Department of Pharmaceutical and Pharmacological Sciences, KU Leuven, Leuven, Belgium; 3 Research Foundation - Flanders (FWO), Brussels, Belgium; 4 Department of Public Health and Primary Care, Academic Center for Nursing and Midwifery, KU Leuven, Leuven, Belgium; 5 Department of Geriatric Medicine, University Hospitals Leuven, Leuven, Belgium; 6 Department of Public Health and Primary Care, Gerontology and Geriatrics, KU Leuven, Leuven, Belgium

**Keywords:** Deprescribing, Tapering, Adverse events, Benzodiazepines, z-drugs

## Abstract

**Background:**

Chronic use of hypnotic agents is prevalent in older adults, who as a result are at increased risk for certain adverse events, such as day-time drowsiness and falls. Multiple strategies to discontinue hypnotics have been tested in geriatric patients, but evidence remains scarce. Hence, we aimed to investigate a multicomponent intervention to reduce hypnotic drug use in geriatric inpatients.

**Methods:**

A before-after study was performed on the acute geriatric wards of a teaching hospital. The before group (= control group) received usual care, while intervention patients (= intervention group) were exposed to a pharmacist-led deprescribing intervention, comprising education of health care personnel, access to standardized discontinuation regimens, patient education and support of transitional care. The primary outcome was hypnotic drug discontinuation at one month after discharge. Secondary outcomes among others were sleep quality and hypnotic use at one and two weeks after enrolment and at discharge. Sleep quality was assessed using the Pittsburgh Sleep Quality Index (PSQI) upon inclusion, two weeks after enrolment and one month after discharge. Determinants for the primary outcome were identified using regression analysis.

**Results:**

A total of 173 patients were enrolled, with 70.5% of patients taking benzodiazepines. Average age was 85 years (interquartile range 81–88.5) and 28.3% were male. A higher discontinuation rate at one month after discharge was observed in favour of the intervention (37.7% vs. 21.9%, *p* = 0.02281). No difference in sleep quality was found between both groups (*p* = 0.719). The average sleep quality was 8.74 (95% confidence interval (CI): 7.98–9.49) and 8.57 (95% CI: 7.75–9.39) in the control and intervention groups respectively. Determinants for discontinuation at one month were: the intervention (odds ratio (OR) 2.36, 95% CI: 1.14–4.99), fall on admission (OR 2.05; 95% CI: 0.95–4.43), use of a z-drug (OR 0.54, 95% CI: 0.23–1.22), PSQI score on admission (OR 1.08, 95% CI: 0.97–1.19) and discontinuation prior to discharge (OR 4.71, 95% CI: 2.26–10.17).

**Conclusions:**

A pharmacist-led intervention in geriatric inpatients was associated with a reduction of hypnotic drug use one month after discharge, without any loss in sleep quality.

**Trial registration:**

ClinicalTrials.gov Identifier: NCT05521971 (retrospectively registered on 29^th^ of August 2022).

## Background

Many older adults incur net harm from using hypnotics. Glass et al. concluded in their meta-analysis that the impact of hypnotics on insomnia in older adults is limited in time and moderate in effect at best [1]. Furthermore, their positive effects seem to be offset by negative outcomes such as falls, fractures and cognitive impairment [2–4]. Geriatric patients are more susceptible to such negative outcomes, in part due to diminished physiological reserve, presence of specific morbidities as well as altered pharmacokinetics and pharmacodynamics [5]. Consequently, the risk of these adverse outcomes might be so high in older adults that the upfront use might not be justified in the management of insomnia [1].

Major indications for hypnotics in geriatric patients are insomnia and anxiety, where these agents are frequently initiated as first-line therapies. While clinical practice guidelines recommend non-pharmacological treatments as first-line option, they are only pursued in a minority of patients, in part because drug therapies are easy to start, patient motivation might be low and access to trained health care professionals might be limited [6–9]. About one in five community dwelling older adults and up to half of all nursing home residents use at least one hypnotic drug in Belgium [10, 11]. While prescribers should proactively attempt to limit the treatment duration, e.g., to four weeks, chronic use still frequently occurs [6–9]. Hence, hypnotic overuse in geriatric patients can be understood from the high prevalence of insomnia, but also from the easy initiation of hypnotics and not defining or following up on an explicitly defined therapy duration. In many patients, there is a lack of a clear indication for prolonging the hypnotic prescription according to the current clinical practice guidelines [12].

Harm due to hypnotics can be mitigated by discontinuing the therapy altogether or by at least lowering the dose. Numerous strategies to safely and effectively deprescribe chronic hypnotic drug use in older adults have been investigated [13]. Successful discontinuation might however be impeded by a lack of suitable alternatives, therapeutic inertia, an overabundance of tapering regimens, the presence of dependence and fear of developing withdrawal symptoms upon discontinuation [14]. Importantly, discontinuation of benzodiazepines appears to be feasible and safe in older people, at least in a strict trial setting. Success seems to vary according to the type of intervention, setting and population taking into account that few data have been collected in the subgroup of geriatric inpatients [13, 15]. In EMPOWER, a low-cost and feasible intervention, which mainly consisted of a brochure, was associated with more deprescribing in a community patient sample [16]. Petrovic et al. successfully piloted a semi-abrupt discontinuation method in geriatric inpatients with success [17]. Yet, their conclusions were limited owing to the small and selected patient sample. The overall evidence base remains subpar regarding interventions in geriatric patients during hospital stay and in the post-discharge phase [13, 18].

We hypothesized that a pharmacist-led intervention comprising a medication review, the availability of a rapid discontinuation regimen and education of both patients and health care professionals might increase the likelihood of hypnotic drug discontinuation in geriatric patients at one month after discharge. Therefore, we aimed to test an multi-component pharmacist-led intervention in a before-after study.

## Methods

### Study design & setting

We conducted a monocentric prospective before-after study at the acute geriatric wards of the University Hospitals Leuven (UZ Leuven) in Leuven, Belgium. In the before group patients received usual care (= control group), which consisted of comprehensive geriatric care coordinated by a geriatrician. Patients in the after group were, on top of the usual care, exposed to the pharmacist-led multicomponent intervention (= intervention group). Written informed consent was provided by the patient; or in case of impaired cognition by their caregiver. Ethical approval was obtained from the Ethics Committee UZ/KU Leuven (S59134). Our study adhered to the Declaration of Helsinki as well as to the CONSORT guidelines regarding the reporting of clinical trials [19].

### Study participants

All consecutive patients aged ≥ 75 years admitted to the acute geriatric wards of UZ Leuven with documented chronic use of a hypnotic drug for insomnia, anxiety or an undefined reason were eligible for inclusion. In a first period, patients were included in the control group. Afterwards, when the intervention was implemented, participants were enrolled in the intervention group.

Hypnotics were defined as benzodiazepines and Z-drugs. Chronic hypnotic drug use was defined as hypnotic use for at least five days a week during a minimum of four consecutive weeks. The four week threshold was defined to separate short from long-term use [9]. Patients were excluded if they met any of the following criteria: concomitant use of multiple benzodiazepines and/or Z-drugs, discontinuation of the hypnotic drug prior to enrolment, estimated discharge from the hospital within 72 h of admission, no command of the Dutch language, severe psychiatric or neurological disease (e.g., bipolar disorder, epilepsy or dystonia) in the opinion of the treating physician, a severe acute medical condition and end-of-life care. Study participants who died during their hospital stay were excluded from the analysis as their medication at discharge could not be evaluated. In case of any readmission, only the first admission was included in the analysis.

### Usual care and intervention

In the control group, all patients received comprehensive geriatric care without any systematic clinical pharmacist involvement regarding deprescribing of hypnotics. Hence, the control group received usual care.

In the intervention group, a pharmacist-led intervention was implemented on the geriatric unit comprising the four following components: 1) education of health care personnel; 2) access to standardized discontinuation regimens; 3) patient education; 4) support of transitional care. First, educational sessions on the study, the actual intervention but also on non-pharmacological support measures were provided to the physicians and nursing staff to increase awareness on the importance of hypnotic deprescribing in geriatric inpatients. A flyer was disseminated with information on the actual intervention and supporting measures. Secondly, discontinuation regimens were developed for benzodiazepines and Z-drugs. Clinical pharmacists advised prescribers to implement these regimens in included intervention patients, but prescribers were free to choose whether or not to actually use them. The regimens were derived from the regimen used by Petrovic et al. and encouraged a switch from any benzodiazepine to lorazepam 1 mg once daily (OD) for one week followed by complete discontinuation [17]. Z-drugs were switched to zolpidem 5 mg OD for one week followed by complete discontinuation as well. If deemed necessary, a *pro re nata* regimen of lorazepam 1 mg or zolpidem 5 mg for one additional week was prescribed respectively. These approaches were standardized to facilitate uptake among the prescribers. The discontinuation regimens were incorporated into the hospital’s computerized physician order entry (CPOE) allowing physicians to easily alter the electronic prescriptions. Furthermore, a clinical decision support system (CDSS) provided additional support as it alerted physicians whenever a patient was admitted to an acute geriatric ward with evidence of prolonged use of benzodiazepine and/or Z-drug in their pre-admission medication list. The CDSS then guided the physician to the appropriate discontinuation regimen in the CPOE. Thirdly, patients and their caretakers were informed by the clinical pharmacist both upon enrolment as well as at discharge about potential side effects of hypnotic drugs and the possibility of worsening sleep in the first days after discontinuation. Patients were also provided with information about any discontinuation attempt that was initiated during hospital stay. If a discontinuation regimen with a different hypnotic was implemented, patients were invited to return the leftovers to their community pharmacist. Specific patient leaflets were used to facilitate patient education. Upon discharge, patient and caregivers also received a written summary with information on the intervention. Fourthly, the patient’s primary care physician was informed by phone about the patient’s study participation and the patient received a letter to turn over to his/her primary care physician with more detailed information on the intervention. If patients were discharged to a nursing home, nursing home staff was informed about the current status of the patient’s hypnotic drug use by phone.

### Outcomes

The primary outcome was hypnotic drug discontinuation at one month after discharge. Secondary outcomes related to hypnotic drug use were defined as follows: hypnotic drug use at one and two weeks after enrolment and at hospital discharge; the emergency use of hypnotics after any discontinuation attempt during hospital stay. During hospital stay, the incidence of physician-identified delirium was assessed together with emergency use of antipsychotics. Sleep quality was determined using the Pittsburgh Sleep Quality Index (PSQI) upon inclusion, fourteen days after enrolment and one month after discharge [20]. The PSQI concerns a validated questionnaire where a higher score signifies a worse sleep quality; a total score of 5 or more indicates poor sleep quality. We also asked about the occurrence of falls up to one month after discharge by phone. Finally, we explored determinants for post-discharge hypnotic discontinuation.

### Data collection

The following patient characteristics were obtained from the electronic health record upon admission: age, sex, length of stay, presence of cognitive impairment (including the most recent Mini-Mental State Examination (MMSE) score [21], if available), number of preadmission chronic medications, documentation of delirium prior to admission and whether the reason for admission was fall-related. The latter was defined as no fall, fall without injury, fall with minor injury (without a need for medical intervention) or fall with major injury (e.g., severe hematoma, loss of consciousness, fractures, major cuts). Following data on hypnotic drugs were collected: molecule, indication, equivalent daily dose of lorazepam or zolpidem for benzodiazepines or Z-drugs, respectively. Dose equivalence was estimated using the online dose conversion table of the Belgian Centre for Pharmacotherapeutic Information. The use of other hypnotic drugs such as trazodone, mirtazapine or a barbiturate was also documented. Hypnotic drug use was assessed in this manner upon inclusion, seven and fourteen days after inclusion, upon discharge and one month after discharge. Sleep quality was assessed using the PSQI during an interview with the patient and the caregiver.

### Sample size estimation

The study was designed to detect a 22.5% difference using a chi square test for the primary outcome between the control and intervention groups, taking into account a type I error of 5% and a type II error of 20%. We hypothesized—based on the Petrovic study (i.e., 80 vs. 50% discontinuation rate)—that an absolute reduction of 20% would be feasible [17]. Furthermore, we took into account a potential 2.5% additional impact to account for potential time-related interventions (e.g., hospital-wide deprescribing campaigns). Assuming a 50% medication stop in the control group, a minimal sample size of 73 patients in each group was needed. The baseline discontinuation rate was chosen conservatively as it increases the estimated sample size for a similar absolute difference. To account for a 10% potential loss due to attrition, we aimed to enrol at least 160 study participants in total.

### Statistical analysis

Descriptive statistics were applied to summarize patient characteristics. Normality was evaluated visually, using histograms and QQ-plots. Normally distributed variables were reported using means and standard deviations. Non-normally distributed continuous variables were characterized by medians and interquartile range (IQR, represented as Q1-Q3). Categorical variables were depicted as number of cases and proportions (n, %).

A chi square test was used for the analysis of the primary outcome. Determinants for hypnotic drug discontinuation one month after discharge were evaluated using multivariable logistic regression. An unadjusted logistic regression was performed for the primary outcome with allocation as the single covariate. Afterwards, an explorative model was derived with covariates selected by backwards elimination based on the Akaike information criterion (AIC) value and taking into account missingness (< 5%) and a plausible link with the primary outcome. The most informative model was the one with lowest AIC using a threshold of AIC > 2. The following covariates were included: number of chronic medications prior to admission, presence of cognitive impairment, sex, history of delirium, type of hypnotic on admission, the PSQI score on admission, fall as reason for admission, intervention (vs. control) and hypnotic drug discontinuation at discharge. Collinearity was excluded prior to including the covariates in the model. The residuals distribution of the final model was confirmed to be approximately normal. This approach provided odds ratios (OR) with 95% confidence intervals (95% CI).

Linear mixed modelling was used to assess the sleep quality. Time (upon admission, at discharge and 1 month after discharge) and study group (control vs. intervention) were used as fixed factors; random effects pertained to individual study subjects. If no significant interaction between time and group was observed, main effects results were reported.

A two sided *p*-value < 0.05 was considered to be statistically significant. All analyses were performed using R (4.1.3, R Core Team, Vienna, Austria) in the RStudio environment (2022.07.1 + 554, Boston, MA, USA). ‘MASS’ was used to develop the multivariable regression model. Packages ‘lmerTest’ and ‘emmeans’ were used to derive the linear mixed model.

## Results

### Patient flow and baseline characteristics

**Fig. 1:**
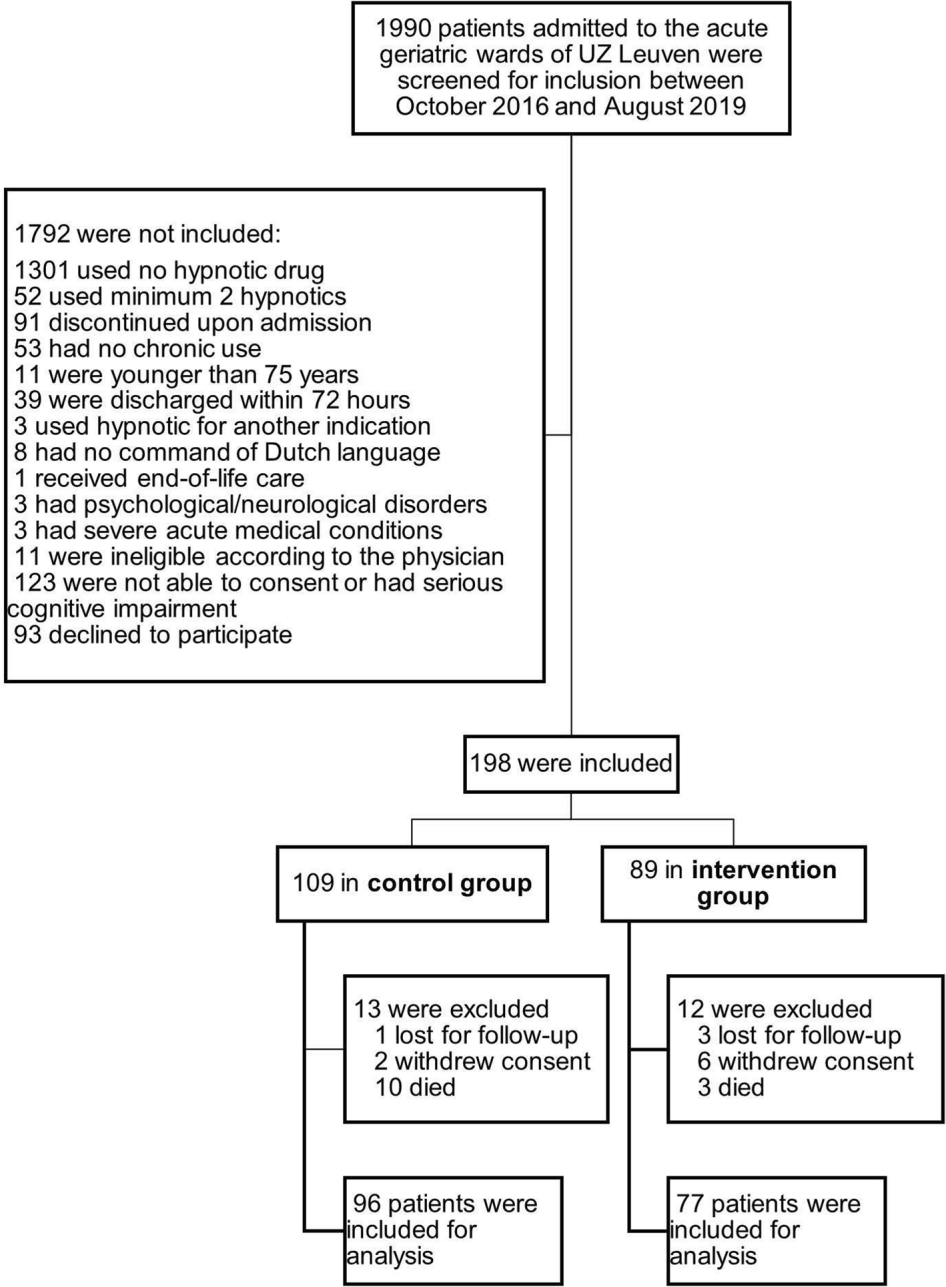
Participant flow

Between October 2016 and August 2019, the study ran for 10 (control group) and 11 months (intervention group). During the study period, 1990 patients were admitted to the acute geriatric wards of UZ Leuven and were screened for inclusion. A major reason for not being enrolled was no use of hypnotics upon admission to the ward (*n* = 1301). In total, 198 patients were included in the study and 25 were excluded from the study after enrolment. Finally, data on 173 patients were used in the final analysis, with 96 and 77 in the control and intervention group respectively. Patient flow in the study has been summarized in Fig. 1.

Patients were aged on average 85 years (IQR 81–88.5) and 28.3% were male. Patients used 10 drugs (IQR 7–13) prior to admission. About three quarters of all patients used a benzodiazepine, which included twelve different active compounds. Lorazepam was used most frequently (43/173, 24.9%) followed by lormetazepam (36/173 cases, 20.8%). Zolpidem was the mainstay Z-drug, in all but one patient, who used zopiclone. Insomnia was the main indication for hypnotic drug use and was present in 155/173 (89.6%) patients.

Cognitive impairment was documented in 19/173 (11%) of all patients. The median of the last reported MMSE value was 24/30 (IQR 22–26) in 55 patients and 24/30 (IQR 20–27) in 54 patients of the control and intervention groups respectively. The proportion of patients who already experienced a delirium before admission was 8.3% and 19.5% respectively for the control and intervention groups. A fall as reason for admission was registered more frequently in the intervention group, where 25 patients (32.5%) were admitted because of a fall compared to 26% in the control group. The average length of stay was 11 days (IQR 7–15.5). Baseline patient data have been summarized in Table 1.

**Table 1:** Patient baseline characteristics

Variable	Total study population (*N* = 173)	Control group (*N* = 96)	Intervention group (*N* = 77)
Age in years, median (IQR)	85 (81–88.5)	86 (82–89)	85 (81–88)
Sex n (%)			
Men	49 (28.3)	24 (25)	25 (32.5)
Women	124 (71.7)	72 (75)	52 (67.5)
Cognitive impairment, n (%)	19 (11)	9 (9.4)	10 (13)
Delirium in medical history, n (%)	23 (13.3)	8 (8.3)	15 (19.5)
MMSE, median (IQR)	24 (20,3–26) in 100 patients	24 (22–26) in 55 patients	24 (20–27) in 45 patients
Fall on admission, n (%)	50 (28.9)	25 (26)	25 (32.5)
No injury	16 (9.2)	8 (8.3)	8 (10.4)
Minor injuries	21 (12.1)	8 (8.3)	13 (16.9)
Major injuries	13 (7.5)	9 (9.4)	4 (5.2)
Length of stay (days), median (IQR)	11 (7–15.5)	12 (8–15.8)	9 (7–15.5)
Number of pre-admission medications, median (IQR)	10 (7–13)	9.5 (6.3–12.8)	10 (7–13.5)
Type of hypnotic drug, n (%)			
Benzodiazepine	122 (70.5)	71 (74)	51 (66.2)
Z-drug	51 (29.5)	25 (26)	26 (33.8)
Equivalent daily dose, median (IQR)			
Lorazepam (mg)	2.5 (1.25–4.4)	2.5 (1–4.4)	3.33 (1.3–5)
Zolpidem (mg)	10 (5–10)	10 (5–10)	10 (5–10)
Indication, n (%)			
Insomnia	155 (89.6)	85 (88.5)	70 (90.9)
Anxiety	11 (6.4)	7 (7.3)	4 (5.2)
Insomnia & anxiety	2 (1.2)	1 (1.0)	1 (1.3)
Unknown	4 (2.3)	3 (3.1)	1 (1.3)
Other	1 (0.6)	/	1 (1.3)
Molecule, n (%)			
Alprazolam	18 (10.4)	7 (7.3)	11 (14.3)
Bromazepam	9 (5.2)	5 (5.2)	4 (5.2)
Clonazepam	1 (0.6)	1 (1.0)	/
Clorazepate	1 (0.6)	/	1 (1.3)
Diazepam	4 (2.3)	2 (2.1)	2 (2.6)
Flunitrazepam	1 (0.6)	1 (1.0)	/
Flurazepam	1 (0.6)	1 (1.0)	/
Loprazolam	1 (0.6)	1 (1.0)	/
Lorazepam	43 (24.9)	30 (31.3)	13 (16.9)
Lormetazepam	36 (20.8)	20 (20.8)	16 (20.8)
Nitrazepam	2 (1.2)	1 (1.0)	1 (1.0)
Oxazepam	3 (1.7)	1 (1.0)	2 (2.6)
Prazepam	2 (1.2)	1 (1.0)	1 (1.0)
Zolpidem	50 (28.9)	24 (25.0)	26 (33.8)
Zopiclone	1 (0.6)	1 (1.0)	/
Intake upon admission of			
Trazodone, n (%)	17 (9.8)	6 (6.3)	11 (14.3)
Mirtazapine, n (%)	7 (4)	5 (5.2)	2 (2.6)
Barbiturates, n (%)	0	0	0

*IQR *Interquartile range, *MMSE *Mini-Mental State Examination

### Hypnotic drug outcomes

Discontinuation was initiated in more intervention compared to control patients (81.6% vs. 57.3%) with a higher concomitant uptake of the standardized tapering regimen (66.2% vs 0%), respectively. The hypnotic drug was abruptly discontinued in 17 (17.7%) and 8 (10.4%) in the control vs. intervention groups respectively. A total of 21 (21.9%) patients in the control and 29 (37.7%) patients in the intervention group reported discontinuation of benzodiazepines or Z-drugs one month after discharge. Hypnotic drug use from admission up to one month after discharge has been summarized in Table 2.

**Table 2:** Hypnotic drug use from admission up to one month after discharge

Outcomes	Control group (*N* = 96)	Intervention group (*N* = 77)
**Attempts to reduce the dose of or discontinue the hypnotic during hospital stay**		
Implementation of standardized tapering regimen, n (%)	Not applicable	51 (66.2%)
Any attempt	55 (57.3%)	62 (81.6%)
Abrupt discontinuation	17 (17.7%)	8 (10.4%)
**One week after inclusion**		
Discontinuation n (%)	27 (28.1)	18 (23.4)
Dose reduction, n (%)	27 (28.1)	40 (51.9)
**Two weeks after inclusion**		
Discontinuation, n (%)	25 (26.0)	28 (36.4)
Dose reduction, n (%)	21 (21.9)	19 (24.7)
**Upon discharge**		
Discontinuation, n (%)	34 (35.4)	30 (39.0)
Dose reduction, n (%)	31 (32.3)	29 (37.7)
**One month after discharge**		
Primary outcome: discontinuation of hypnotic drug one month after discharge, n (%)	21 (21.9)	29 (37.7)
Dose reduction, n (%)	22 (22.9)	17 (22.1)

The intervention resulted in a significant reduction in hypnotic drug use one month after discharge (*Χ*^*2*^ (1, *N* = 173) = 5.1826, *p* = 0.02281). The reduction was driven by a reduction in benzodiazepine use (15/96 vs. 23/77) while no impact was seen on Z-drug use (6/96 vs. 6/77). The average estimated dose of lorazepam (control: 2.50 mg (IQR: 1.00–3.3 mg) vs. intervention: 1.25 mg (IQR: 1.00–3.75 mg)) and zolpidem (control: 10 mg (IQR: 5-10 mg) vs. intervention: 5 mg (IQR: 5-10 mg)) did not differ among those who still were prescribed these agents at one month after discharge.

The intervention was associated with an OR of 2.16 (95% CI: 1.11–4.25) for the primary outcome in an unadjusted analysis. The AIC-based backwards regression approach found the lowest AIC for the following five covariates: fall on admission, baseline PSQI score, drug discontinuation at discharge, type of hypnotic drug and the intervention. The intervention remained significantly associated with the primary outcome even after adjusting for these confounders. Coefficients are summarized in Table 3.

**Table 3:** Explorative multivariable logistic regression analysis for hypnotic drug discontinuation one month after discharge

Variable	Odds ratio	Lower margin CI (2.5%)	Upper margin CI (97.5%)	*p*-value
Intervention (vs. control)	2.36	1.14	4.99	0.022
Fall on admission (yes vs. no)	2.05	0.95	4.43	0.066
Type of hypnotic drug on admission (Z-drug vs. benzodiazepine)	0.54	0.23	1.22	0.149
PSQI score on admission	1.08	0.97	1.19	0.159
Hypnotic discontinued prior to discharge (yes vs. no)	4.71	2.26	10.17	< 0.001

*CI* Confidence interval, *PSQI* Pittsburgh Sleep Quality Index

### Safety

No signal of harm was noted with similar incidences of emergency hypnotic use in both groups (control vs. intervention: 7/96 vs. 6/77), emergency antipsychotic use (3/96 vs. 1/77) and occurrence of delirium (10/96 vs. 9/77) during hospital stay. Post-discharge falls were also documented in comparable numbers of patients with 10/96 and 7/77 in the control and intervention groups respectively. Only one injurious fall was documented during follow-up after discharge in a single patient in the control group.

### Sleep quality

The linear mixed model analysis found no interaction between time and group (*p* = 0.487). Neither group (*p* = 0.719), nor time (*p* = 0.872) was associated with any relevant change in PSQI scores. The estimated marginal means for the PSQI did not differ between the two groups (control: 8.74, 95% CI: 7.98–9.49; intervention: 8.57, 95% CI: 7.75–9.39).

## Discussion

We found that a pharmacist-led intervention effectively and safely reduced the use of hypnotics in geriatric patients one month after discharge. We observed a 15.8% surplus reduction of hypnotics compared to usual care, which coincided with an approximately doubling of the odds of hypnotic drug discontinuation. Our result was driven exclusively by the impact of the intervention on the class of benzodiazepines, whereas no meaningful effect on Z-drugs was observed. Poignantly, differences in discontinuation, while already present at discharge, only further increased thereafter, in part due to substantial reinitiation of the hypnotic in the control group. This finding might be explained by the transitional care component in the intervention group, providing a better support network for the patients. In absolute terms, 7 patients would have to be exposed to the intervention for one additional discontinuation at one month after discharge. We showed that benzodiazepine withdrawal was feasible in geriatric patients with a discontinuation rate that fell within the range of 27% to 80% as published by Reeve et al. [13]. In that sense, our study adds to the current evidence on deprescribing during hospital stay [14, 15]. Particularly, such study findings are notably more important for geriatric inpatients, historically a disenfranchised group regarding clinical trials [13, 22].

We believe our results are valid owing to the following reasons. Firstly, our study was sufficiently powered. Secondly, we tested our intervention in a real life geriatric setting, with few exclusion criteria, adding to the external validity. As a result, our study is among the largest prospective investigations on hypnotic drug use in geriatric inpatients. Thirdly, we monitored inpatients up to one month after discharge with few drop-outs. Lastly, the pharmacist intervention itself was easy to implement and remained associated with discontinuation even after adjusting for potential confounders. In that regard, it is worth mentioning that stopping the hypnotic prior to discharge was the largest determinant for successful discontinuation one month later. This was possible in 39% of the intervention patients.

Yet, some limitations need to be taken into account. Firstly, the study was monocentric and quasi-experimental as we relied on an uncontrolled before-after design. While this approach might be not as robust as the randomized controlled trial design, we decided against the latter given the risk of contamination bias. Many of the intervention components were implemented across the entire geriatric unit (e.g., staff education and incorporation of tapering regimens in the CPOE). We did not perform an interrupted time series analysis given limited resources, as this would prolong the study period substantially. Secondly, we were only able to provide follow-up up to one month after discharge. Initially, we planned a longer follow-up period of twelve months. Members of the Ethics Committee were concerned however about this duration as they felt the intervention might then potentially interfere with the physician–patient relationship. For this reason, we limited follow-up to one month after discharge. This period would not interfere substantially with primary care, and would still provide us with sufficient time to evaluate the impact of our intervention. We are however unable to make statements beyond one month after discharge and cannot exclude a higher reuptake of hypnotics thereafter. Thirdly, given the multicomponent nature of our intervention, we could not identify which actual components were most strongly associated with the primary outcome. Fourthly, given the before-after design, a selection bias might also have been present. Intervention patients might have been more motivated to participate as they might have been more inclined to discontinue their hypnotic drugs. However, we tried to mitigate selection bias by explicitly stating that patients were in no way obligated to discontinue their hypnotic drug. Finally, to improve the internal validity of our study, we limited the study specifically to those patients who only used one hypnotic at the time, thereby excluding 52 patients from study participation. This limits our ability to provide recommendations on deprescribing for those who take more than one hypnotic drug.

Our data are largely in line with prior published findings, bearing in mind that previous studies in geriatric inpatients are limited. The majority of evidence hails from other patient groups and care settings [13, 15]. Some argue whether hospital stay is even the right time for deprescribing, given the acute nature of the stay and the many medication alterations over the course of a few days. Conversely, an important advantage is that the multidisciplinary setting, the close clinical monitoring and the presence of bed-side nursing staff all allow for a semi-abrupt approach, which is less likely to succeed outside of the hospital setting [23, 24]. Therefore, we fully agree with Petrovic et al. that hospital stay provides ample opportunity to deprescribe, particularly in geriatric inpatients in whom there frequently is a clinical event associated with prior use of an hypnotic (e.g., a fall) leading to the unplanned admission [17]. Importantly, we do not make any claims that a comparable semi-abrupt intervention should be implemented in general practice without close monitoring. Further study is still needed on the efficacy and more importantly on the safety of such a rapid approach, prior to rolling out our multicomponent intervention in general practice and/or healthier geriatric outpatients.

Our pharmacist-led intervention was centred around transitional care, education and a standardized tapering approach. A standardized tapering approach has been found to be more effective than usual care [16, 25–27]. Several regimens have been studied, ranging from an abrupt stop to more prolonged discontinuation regimens such as lowering the dose by 10–25% each two weeks [13]. Given the limited duration of hospital stay, we applied a fast withdrawal approach, mostly influenced by the previous work of Petrovic et al. First, Petrovic et al. conducted an uncontrolled interventional study in which a fast withdrawal scheme was tested for chronic benzodiazepine users admitted to an acute geriatric ward (*n* = 49) [28]. For one week, the drug was replaced by either trazodone 50 mg or lormetazepam 1 mg. Afterwards the replacement agent was discontinued. The authors concluded that this was effective in 67.9% of the patients without any signal of harm. Subsequently, the same group conducted a small double-blind randomized controlled trial (RCT) on a short-term replacement therapy versus placebo (*n* = 40) [17]. Enrolled geriatric inpatients received either 1 mg lormetazepam or placebo for one week. In the group that received lormetazepam, the success rate was significantly higher compared to the placebo group (80% vs. 50%, *p* < 0.05). Our study results are largely in line with those of Petrovic et al*.* and could be seen as an external validation of their initial experiences: a short taper is possible and safe in geriatric inpatients and is associated with reduced use after discharge.

Furthermore, we made sure to actively involve primary care physicians prior to discharge in the intervention group. We believe this to be essential to ensure post-discharge success. In our study, a specific discontinuation letter (including explicit instructions) was provided in addition to the discharge letter. Furthermore, the primary care physician was contacted by phone at discharge by the clinical pharmacist to further promote the withdrawal regimen that was implemented during hospitalization or at discharge. Without primary care involvement, the intervention is limited to hospital stay and mostly a function of the motivation of the patients and their caretakers. Bourgeois et al. conducted a cohort study where a letter was sent to the primary care physician requesting them to initiate discontinuation of hypnotic agents in eligible nursing home patients who were known chronic benzodiazepine or Z-drug users. The letter included a standardized regimen but physicians were allowed to discontinue benzodiazepines according to their own discretion [29]. Discontinuation was attempted in 38 out of 135 residents with a 8-month success rate of 66%. The major reason for refusal was patients not being motivated. In the study of Midlov et al., education outreach was provided by a pharmacist and a physician who visited primary care physicians to inform them about the adverse effects of benzodiazepines and psychotropic drugs. One year post-intervention, a decrease in benzodiazepine prescribing (26.6%) was found compared to control (*p* < 0.05) [30]. Both investigations show the importance of actively involving primary care, particularly over the long term.

We aimed to actively educate and motivate patients regarding their own hypnotic drug use. We discussed the intervention face-to-face with both patients as well as caretakers and provided additional materials. Any patient involvement should preferably be in person, but a written component might be sufficient to promote hypnotic drug deprescribing. For example, Vicens et al*.* found no difference between verbal in-person education and written instructions from the primary care physician [26]. This was further corroborated by the EMPOWER data where Tannenbaum et al*.* showed that empowerment via a elaborated booklet led to a substantial reduction in hypnotic drug use at six months after inclusion [16]. The same authors later confirmed the validity of this approach in the D-PRESCRIBE trial where they observed similar findings, also related to other (potentially inappropriate) drug classes. Gorgels et al*.* examined the impact of a letter sent by primary care physicians to chronic users of benzodiazepines [31, 32]. After six months, there was a decrease in the number of benzodiazepine prescriptions in the intervention compared to the control group (24% versus 5%, *p* < 0.01). At 21 months, a larger reduction of benzodiazepine prescriptions was observed in the interventional group versus control (26% versus 9%), supporting the persistence of the letter-based intervention. Importantly, such investigations have not been replicated in our patient population who are more frail and about 20 to 25 years older on average.

The sleep quality was suboptimal at baseline and remained so during the entire study duration. This means that overall sleep quality translated to a ‘miserable’ sleep according to the validated PSQI [20]. While there was no impact of the intervention on sleep quality, this might be considered to be reassuring. There was no signal of rebound insomnia in the intervention group, given similar PSQI values between both groups over time and the absence of increased hypnotic reinitiation among intervention patients. Compared to the RCT of Petrovic et al., our general results seem to overlap when taking into account the entire study duration [17]. Discontinuing chronically used hypnotics was not associated with worsened sleep. Furthermore, the PSQI score at baseline was retained as an independent determinant for a higher odds of hypnotics discontinuation at one month after discharge. This further adds to the overall safety of our approach, in that it does not worsen sleep quality or is not less likely to succeed in those with higher (i.e., worse) baseline scores.

Yet, some major questions still remain on how to best approach hypnotics in geriatric inpatients. This study is already a step in the right direction compared to our previous experiences where we were unable to impact hypnotic drug prescribing at discharge [33]. Given the currently limited resources, we did not collect qualitative data among patients and caretakers, precluding us from providing any statements on their viewpoints regarding sleep quality, use of hypnotics and the value of deprescribing. Furthermore, the current study was only limited to one month follow-up. Importantly, this deprescribing intervention has now been incorporated in the ASPIRE intervention, where it will be part of a broader approach to improve medication use and clinical outcome [34]. ASPIRE concerns an ongoing RCT on our acute geriatric wards where the impact of a multifaceted pharmacist intervention will be compared to usual geriatric care on the time to a first unplanned hospital revisit with a follow-up of six months after discharge. All intervention components from this describing study have been adopted except education of prescribers to avoid contamination bias. If ASPIRE is found to be positive, our deprescribing intervention might find a broader uptake given the perceived importance of deprescribing and the availability of ward-based pharmacists to support implementation [35]. Additionally, we eagerly await the findings of the EU-funded (HORIZON-HLTH-2021-CARE-05) BE-SAFE project, coordinated among others by Spinewine and colleagues, which will also focus on hypnotic deprescribing of in older adults.

In sum, our investigation adds another option to the breadth of interventions to reduce the burden of hypnotics among older adults. Based on our findings, we recommend to systematically evaluate hypnotic use in geriatric inpatients and promote the use of our intervention. To this end, a multidisciplinary care team should coordinate this multicomponent deprescribing effort, involving trained clinical pharmacists [36, 37]. This team should oversee correct patient selection as well as monitor the safety of the semi-abrupt discontinuation. The most important step of the entire intervention however is the first one, to actually attempt to reduce hypnotics.

## Conclusion

A pharmacist-led intervention in geriatric inpatients was found to be associated with a reduction of hypnotic drug use one month after discharge, without any signal of harm.

## Data Availability

All data generated or analysed during the current study are available from the corresponding author on reasonable request (via e-mail).

## Abbreviations

AIC: Akaike information criterion
CDSS: Clinical decision support system
CPOE: Computerized physician order entry
CI: Confidence interval
IQR, represented as Q1-Q3: Interquartile range
MMSE: Mini-Mental State Examination
OR: Odds ratios
OD: Once daily
PSQI: Pittsburgh Sleep Quality Index

## References

[1] Glass J, Lanctot KL, Herrmann N, Sproule BA, Busto UE (2005). Sedative hypnotics in older people with insomnia: meta-analysis of risks and benefits. BMJ.

[2] Brandt J, Leong C (2017). Benzodiazepines and Z-Drugs: An Updated Review of Major Adverse Outcomes Reported on in Epidemiologic Research. Drugs R&D.

[3] Hanlon JT, Horner RD, Schmader KE, Fillenbaum GG, Lewis IK, Wall WE, Landerman LR, Pieper CF, Blazer DG, Cohen HJ (1998). Benzodiazepine use and cognitive function among community-dwelling elderly. Clin Pharmacol Ther.

[4] Treves N, Perlman A, Kolenberg Geron L, Asaly A, Matok I (2018). Z-drugs and risk for falls and fractures in older adults-a systematic review and meta-analysis. Age Ageing.

[5] Shi S, Klotz U (2011). Age-related changes in pharmacokinetics. Curr Drug Metab.

[6] Qaseem A, Kansagara D, Forciea MA, Cooke M, Denberg TD (2016). Management of Chronic Insomnia Disorder in Adults: A Clinical Practice Guideline From the American College of Physicians. Ann Intern Med.

[7] Katzman MA, Bleau P, Blier P, Chokka P, Kjernisted K, Van Ameringen M, Antony MM, Bouchard S, Brunet A, Flament M (2014). Canadian clinical practice guidelines for the management of anxiety, posttraumatic stress and obsessive-compulsive disorders. BMC Psychiatry.

[8] Sateia MJ, Buysse DJ, Krystal AD, Neubauer DN, Heald JL (2017). Clinical Practice Guideline for the Pharmacologic Treatment of Chronic Insomnia in Adults: An American Academy of Sleep Medicine Clinical Practice Guideline. J Clin Sleep Med.

[9] Riemann D, Baglioni C, Bassetti C, Bjorvatn B, Dolenc Groselj L, Ellis JG, Espie CA, Garcia-Borreguero D, Gjerstad M, Goncalves M (2017). European guideline for the diagnosis and treatment of insomnia. J Sleep Res.

[10] Petein C, Spinewine A, Henrard S (2021). Trends in benzodiazepine receptor agonists use and associated factors in the Belgian general older population: analysis of the Belgian health interview survey data. Ther Adv Psychopharmacol.

[11] Bourgeois J, Elseviers MM, Azermai M, Van Bortel L, Petrovic M, Vander Stichele RR (2012). Benzodiazepine use in Belgian nursing homes: a closer look into indications and dosages. Eur J Clin Pharmacol.

[12] Petrovic M, Mariman A, Warie H, Afschrift M, Pevernagie D (2003). Is there a rationale for prescription of benzodiazepines in the elderly? Review of the literature. Acta Clin Belg.

[13] Reeve E, Ong M, Wu A, Jansen J, Petrovic M, Gnjidic D (2017). A systematic review of interventions to deprescribe benzodiazepines and other hypnotics among older people. Eur J Clin Pharmacol.

[14] Sawan M, Reeve E, Turner J, Todd A, Steinman MA, Petrovic M, Gnjidic D (2020). A systems approach to identifying the challenges of implementing deprescribing in older adults across different health-care settings and countries: a narrative review. Expert Rev Clin Pharmacol.

[15] Soni A, Thiyagarajan A, Reeve J (2023). Feasibility and effectiveness of deprescribing benzodiazepines and Z-drugs: systematic review and meta-analysis. Addiction.

[16] Tannenbaum C, Martin P, Tamblyn R, Benedetti A, Ahmed S (2014). Reduction of inappropriate benzodiazepine prescriptions among older adults through direct patient education: the EMPOWER cluster randomized trial. JAMA Intern Med.

[17] Petrovic M, Pevernagie D, Mariman A, Van Maele G, Afschrift M (2002). Fast withdrawal from benzodiazepines in geriatric inpatients: a randomised double-blind, placebo-controlled trial. Eur J Clin Pharmacol.

[18] Pollmann AS, Murphy AL, Bergman JC, Gardner DM (2015). Deprescribing benzodiazepines and Z-drugs in community-dwelling adults: a scoping review. BMC Pharmacol Toxicol.

[19] Schulz KF, Altman DG, Moher D (2010). CONSORT 2010 statement: updated guidelines for reporting parallel group randomised trials. BMJ.

[20] Buysse DJ, Reynolds CF, Monk TH, Berman SR, Kupfer DJ (1989). The Pittsburgh Sleep Quality Index: a new instrument for psychiatric practice and research. Psychiatry Res.

[21] Braekhus A, Laake K, Engedal K (1992). The Mini-Mental State Examination: identifying the most efficient variables for detecting cognitive impairment in the elderly. J Am Geriatr Soc.

[22] Sibille FX, Spinewine A, Zerah L, Maljean L, Schoevaerdts D, de Saint-Hubert M (2022). Current practice in benzodiazepine receptor agonists deprescribing on acute geriatric wards: a cohort study. BMC Geriatr.

[23] Borne R, Cumbler E, Glasheen JJ (2011). Reducing polypharmacy: is hospitalization the right time?. Arch Intern Med.

[24] Petrovic M, Somers A, Onder G (2016). Optimization of Geriatric Pharmacotherapy: Role of Multifaceted Cooperation in the Hospital Setting. Drugs Aging.

[25] Vicens C, Fiol F, Llobera J, Campoamor F, Mateu C, Alegret S, Socias I (2006). Withdrawal from long-term benzodiazepine use: randomised trial in family practice. Br J Gen Pract.

[26] Vicens C, Bejarano F, Sempere E, Mateu C, Fiol F, Socias I, Aragones E, Palop V, Beltran JL, Pinol JL (2014). Comparative efficacy of two interventions to discontinue long-term benzodiazepine use: cluster randomised controlled trial in primary care. Br J Psychiatry.

[27] Oude Voshaar RC, Mol AJ, Gorgels WJ, Breteler MH, van Balkom AJ, van de Lisdonk EH, Kan CC, Zitman FG (2003). Cross-validation, predictive validity, and time course of the Benzodiazepine Dependence Self-Report Questionnaire in a benzodiazepine discontinuation trial. Compr Psychiatry.

[28] Petrovic M, Pevernagie D, Van Den Noortgate N, Mariman A, Michielsen W, Afschrift M (1999). A programme for short-term withdrawal from benzodiazepines in geriatric hospital inpatients: success rate and effect on subjective sleep quality. Int J Geriatr Psychiatry.

[29] Bourgeois J, Elseviers MM, Van Bortel L, Petrovic M, Vander Stichele RH (2014). Feasibility of discontinuing chronic benzodiazepine use in nursing home residents: a pilot study. Eur J Clin Pharmacol.

[30] Midlov P, Bondesson A, Eriksson T, Nerbrand C, Hoglund P (2006). Effects of educational outreach visits on prescribing of benzodiazepines and antipsychotic drugs to elderly patients in primary health care in southern Sweden. Fam Pract.

[31] Gorgels WJ, Oude Voshaar RC, Mol AJ, van de Lisdonk EH, van Balkom AJ, van den Hoogen HJ, Mulder J, Breteler MH, Zitman FG (2005). Discontinuation of long-term benzodiazepine use by sending a letter to users in family practice: a prospective controlled intervention study. Drug Alcohol Depend.

[32] Gorgels WJ, Oude Voshaar RC, Mol AJ, van de Lisdonk EH, van Balkom AJ, Breteler MH, van den Hoogen HJ, Mulder J, Zitman FG (2006). Predictors of discontinuation of benzodiazepine prescription after sending a letter to long-term benzodiazepine users in family practice. Fam Pract.

[33] Van der Linden L, Decoutere L, Walgraeve K, Milisen K, Flamaing J, Spriet I, Tournoy J (2017). Combined Use of the Rationalization of Home Medication by an Adjusted STOPP in Older Patients (RASP) List and a Pharmacist-Led Medication Review in Very Old Inpatients: Impact on Quality of Prescribing and Clinical Outcome. Drugs Aging.

[34] Hias J, Hellemans L, Laenen A, Walgraeve K, Liesenborghs A, De Geest S, Luyten J, Spriet I, Flamaing J, Van der Linden L (2022). The effect of a trAnSitional Pharmacist Intervention in geRiatric inpatients on hospital visits after dischargE (ASPIRE): Protocol for a randomized controlled trial. Contemp Clin Trials.

[35] Hias J, Van der Linden L, Walgraeve K, Lemper JC, Hellemans L, Spriet I, Tournoy J (2022). Optimizing pharmacotherapy on geriatric hospital units in Belgium - a national survey. Acta Clin Belg.

[36] Daunt R, Curtin D, O'Mahony D (2023). Polypharmacy stewardship: a novel approach to tackle a major public health crisis. Lancet Healthy Longev.

[37] Van der Linden L, Hias J, Walgraeve K, Flamaing J, Tournoy J, Spriet I (2020). Clinical Pharmacy Services in Older Inpatients: An Evidence-Based Review. Drugs Aging.

